# A Novel Insight into the Role of PLA2R and THSD7A in Membranous Nephropathy

**DOI:** 10.1155/2021/8163298

**Published:** 2021-07-14

**Authors:** Pingna Zhang, Weijun Huang, Qiyan Zheng, Jingyi Tang, Zhaocheng Dong, Yuhua Jiang, Yuning Liu, Weijing Liu

**Affiliations:** ^1^Renal Research Institution of Beijing University of Chinese Medicine, Beijing, China; ^2^Key Laboratory of Chinese Internal Medicine of Ministry of Education and Beijing, Dongzhimen Hospital Affiliated to Beijing University of Chinese Medicine, Beijing, China; ^3^Institute of Nephrology, and Zhanjiang Key Laboratory of Prevention and Management of Chronic Kidney Disease, Guangdong Medical University, Zhanjiang, China

## Abstract

Membranous nephropathy (MN) is an organ-restricted autoimmune disease mainly caused by circulating autoantibodies against podocyte antigens, including the M-type phospholipase A2 receptor (PLA2R) and thrombospondin domain-containing 7A (THSD7A). Antibodies against PLA2R are present in 70%–80% and against THSD7A in 2% of adult patients, which provides a paradigm shift in molecular diagnosis and management monitoring. Both antigens share some similar characteristics: they are expressed by podocytes and have wide tissue distributions; they are bound by autoantibodies only under nonreducing conditions, and the subtype of most autoantibodies is IgG4. However, the factors triggering autoantibody production as well as the association among air pollution, malignancy, and the pathogenesis of MN remain unclear. In this review, we discuss the similarity between the pathological mechanisms triggered by disparate antigens and their associated diseases. Furthermore, we demonstrated the possibility that PM2.5, malignancy, and gene expression specifically induce exposure of these antigens through conformational changes, molecular mimicry, or increased expression eliciting autoimmune responses. Thus, this review provides novel insights into the pathological mechanism of MN.

## 1. Introduction

Membranous nephropathy (MN) is a common autoimmune glomerular lesion, accounting for approximately 20%–30% of nephrotic syndrome cases in adults, with poor kidney prognosis [1, 2]. MN is caused by the deposition of immune complexes on the outer aspect of the glomerular basement membrane (GBM), which results in the thickening of the glomerular capillary wall, with expansion of the matrix material leading to the formation of spikes. The discovery of the M-type phospholipase A2 receptor (PLA2R) in 2009 [3] and thrombospondin domain-containing 7A (THSD7A) in 2014 [4] as two major autoantigens in idiopathic MN (iMN) has considerably advanced the understanding of the molecular pathogenesis. These major breakthroughs have been quickly translated to clinical diagnosis and treatment monitoring, with anti-PLA2R [5] and anti-THSD7A antibodies being detected in 70%–80%and only 2% [4] of adult patients with iMN, respectively. Anti-PLA2R and anti-THSD7A antibody titers are key biomarkers that reflect disease activity [6], predict prognosis [7–10], and indicate treatment efficacy [11–13].

In recent years, more research has focused on the role of the lung as a potential contributor to autoimmune diseases [14]. The interesting phenomenon that long-term exposure to PM2.5 is closely related to the gradual increase in the morbidity of MN should be considered [15]. Furthermore, approximately 20% of patients with THSD7A-associated MN are diagnosed with malignancy within a median time of 3 months since the diagnosis of MN, indicating that THSD7A-associated MN is related to an increased risk of malignancy. However, many aspects related to the initiation of autoantibody production and the association among air pollution, malignancy, and the pathogenesis of MN remain unclear. In this review, we highlight the similar characteristics between the two autoantigens and their associated diseases, discuss the possible mechanisms of initiation of antibody formation, and offer a novel view of the pathobiology of MN.

## 2. A Model Marker of iMN: PLA2R

### 2.1. The M-Type PLA2R

The landmark discovery of the PLA2R antigen revolutionized our understanding of MN, and research on its structural characteristics paved the way for revealing the molecular pathogenetic mechanisms of MN. In 2009, Beck et al. [3] identified a 185 kD protein in glomerular extracts in approximately 70% of samples from patients with iMN in nonreducing conditions. This protein was identified as PLA2R, a transmembrane glycoprotein that is largely confined to glomerular podocytes rather than other human glomerular cell types in the human kidneys. In addition, the endogenous glomerular expression of PLA2R is not detected in rodent or rabbit glomerular extracts. In the presence of reducing agents, reactivity is lost, suggesting that the antigenic epitope in PLA2R is a conformation and requires specific disulfide bonds. PLA2R is a type I transmembrane receptor and one of the four members of the mannose receptor family in mammals [16, 17]. Similar to the characteristics of the other members of this family, the extracellular portion of PLA2R contains an N-terminal cysteine-rich (CysR or ricin B) domain, a single fibronectin type 2 (FnII) domain, and eight C-type lectin-like domains (CTLDs). In addition, the short cytoplasmic domain of PLA2R contains motifs that allow constitutive endocytic recycling through clathrin-coated pits [18]. PLA2R undergoes endocytic recycling, which may provide a constant supply of accessible PLA2R for the formation of immune complexes at the podocyte membrane. Dong et al. [19] have made great progress in the determination of the structure of human M-type PLA2R by low-temperature electron microscopy. The ectodomain has high internal flexibility and adopts a compact dual-ring-shaped conformation at an acidic pH and an extended conformation at a basic pH. Owing to the expansion of the PLA2R structure at a basic pH, several major epitopes located in the CysR, CTLD1, and CTLD7 domains may be more accessible for the production of different antibodies [20–22]. However, whether this pH-dependent conformation is related to disease severity remains unknown. In a recent study, a transgenic mouse line expressing mouse PLA2R1in podocytes was developed, which may open new avenues to address this problem [23].

### 2.2. PLA2R Epitopes and Spreading

**Figure 1:**
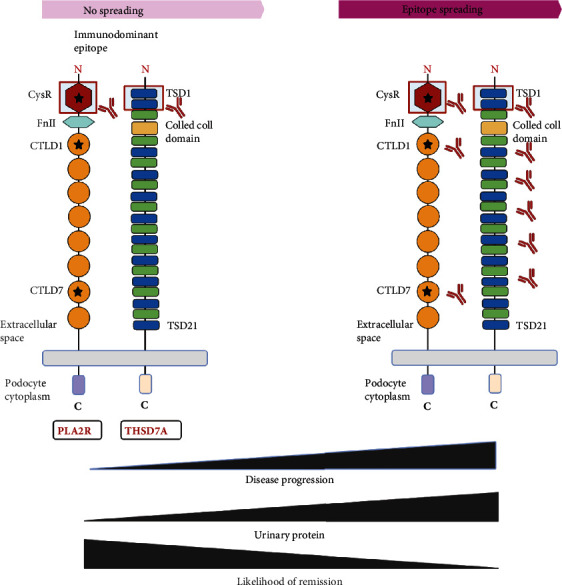
Schematic and epitope spreading of phospholipase A2 receptor (PLA2R) and of thrombospondin type 1 domain-containing 7A (THSD7A). The extracellular part of PLA2R contains an N-terminal cysteine-rich (CysR, or ricin B) domain, a single fibronectin type 2 (FnII) domain, and eight C-type lectin-like domains (CTLDs). The major epitopes are located in the CysR, CTLD1, and CTLD7 domains. THSD7A is a type 1 transmembrane protein with a large extracellular N-terminal region comprising 21 thrombospondin type 1 domains (TSDs) and a coiled coil domain in the extracellular. The predominant target of autoimmunity in two antigens existed in the N-terminal region. Epitope spreading begins with the outermost epitope of the target antigen, and then, other dominant immune epitopes within the molecule or on neighboring molecules are exposed as the disease progresses, which are accompanied by the increase of proteinuria and the decrease of remission.

Over the last decade, the measurement of the levels of circulating PLA2R antibodies has been broadly implemented in clinical practice worldwide for the diagnosis and monitoring of patients with iMN. In addition to the total levels of circulating autoantibodies, a clear picture of major PLA2R epitopes is a prerequisite for understanding the pathogenic mechanism of anti-PLA2R autoantibody binding-induced iMN. Interestingly, two research groups identified B cell epitope-containing domains in the N-terminus of PLA2R with different conclusions because of the stability or folding of the truncated domains of PLA2R in their experimental protocols. Kao et al. [22] showed that the smallest immunodominant epitope in PLA2R containing CysR–FnII–CTLD1. Fresquet et al. [21] narrowed the region to CysR, where the 31 mer peptide blocked most of the autoantibody binding, possibly localizing the humoral epitope to this region. A subsequent study indicated that in addition to CysR, reactive epitopes were present in the CTLD1 and CTLD7 domains. CysR appeared as an immunodominant epitope and was recognized in all patients with circulating anti-PLA2R antibodies (Figure 1) [20].

With further research on autoimmune diseases, PLA2R epitope spreading has been recently identified as a prognostic biomarker to predict outcomes in MN. Epitope spreading, a phenomenon of diversity of epitopes recognized by T and B cells, begins with the outermost epitope of the target antigen (CysR for PLA2R), and then, other dominant immune epitopes (CTLD1 and/or CTLD7 for PLA2R) within the molecule or on neighboring molecules are exposed as the disease progresses, contributing to the expansion of the immune response [24]. As a result, the diversity of antibody repertoire is increased and the overall immune response is enhanced. Experimental evidence indicating that intramolecular epitope spreading might affect the severity of MN in humans has been previously established in Heymann nephritis [25]. Few weeks after the primary immune response, the serum of rats showed reactivity with ligand-binding domain fragments, indicating B cell epitope spreading; furthermore, proteinuria occurred as the epitope spread to further regions in the molecule. In a study, 69 patients with MN whose autoantibody repertoire was confined only to anti-CysR were younger and generally had mild disease activity [20], whereas those with antibodies against all three epitopes tended to be resistant to therapy and had poor renal prognosis. These observations showed that the immune response to PLA2R is an ordered process with prognostic relevance. Of note, the author hypothesized that a second immune hit induces intramolecular epitope spreading. In general, epitope spreading produces a more robust immune response and is more resistant to immunosuppressive treatment [26]. In a prospective cohort of 55 patients treated with rituximab [27], it was necessary to administer higher doses of rituximab to patients with epitope spreading to eliminate the autoantibodies and induce remission. Interestingly, spreaders tended to have higher anti-PLA2R autoantibody titers [28]. Therefore, until clinical testing of anti-PLA2R epitope spreading is routinely available, it seems appropriate that the total titer of anti-PLA2R autoantibodies serves as a surrogate for epitope spreading [29]. Identification of the epitopes will give rise to the development of novel and individualized therapies. Therefore, it is worth exploring the factors initiating epitope spreading.

## 3. The Secondary Marker of iMN: THSD7A

### 3.1. Characteristics of THSD7A and Its Epitopes

The structural characteristics and epitopes of THSD7A have been gradually revealed. In 2014, after PLA2R, Tomas et al. [4] used a combination of glycoproteins purified from glomerular extracts, followed by Western blotting of the extracts under nonreducing conditions to detect antibodies that were reactive with a 250- kD protein in 2.5%–5% of samples from patients with iMN. This protein was identified by mass spectrometry as THSD7A, which is a type 1 transmembrane protein with a large extracellular N-terminal region comprising 21 thrombospondin type 1 (TSP-1) domains, a coiled coil domain, a single-pass transmembrane domain, and a short intracellular C-terminal tail (Figure 1). Seifert et al. [30] and Stoddard et al. [31] showed that these TSP-1 domains are included by alternating TSP-1-like and F-spondin-like domains, both of which consist of three antiparallel peptide strands tightly connected by three disulfide bridges between cysteine residues. Similar to PLA2R, THSD7A is expressed on the basal aspect of foot processes, and the subtype of most anti-THSD7A autoantibodies is IgG4. In addition, the nonreduced form of THSD7A can be exclusively recognized by autoantibodies, suggesting that disulfide bonds determine the antigenic epitope conformation. Compared with PLA2R, the identification of antigenic epitopes in THSD7A is less well defined. THSD7A is predicted to contain multiple epitopes in 18 domains (domains 1–17 and 19) by homology modeling [31], which corresponded well to the domains identified by Seifert et al. [30], except for domains 4 and 5. Additional epitope mapping revealed that the dominant epitope in THSD7A-associated MN is located within the N-terminal TSR1 domain, recognized by autoantibodies in 87% of patient serum samples. Although we have a general understanding of the epitopes in THSD7A, whether epitope spreading could also be a relevant biomarker of disease activity and clinical outcome remains to be determined.

### 3.2. Pathogenicity of Anti-THSD7A Antibodies

**Figure 2:**
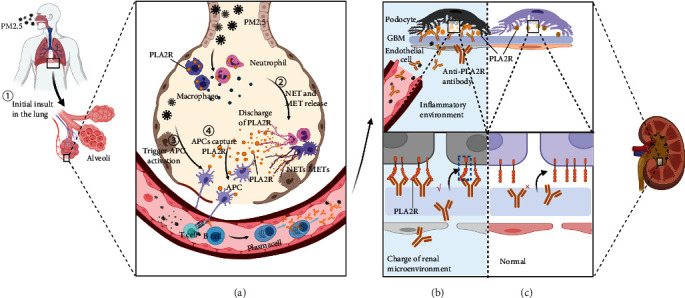
The hypothetical pathogenesis model of the relationship between the tumor and thrombospondin domain-containing 7A- (THSD7A-) associated membranous nephropathy (MN). (a) In theory, THSD7A acts as a potential tumor-associated antigen that is overexpressed by cancerous tissue allowing regional exposure of pathogenic epitopes of the autoantigen, which contributes to the production of anti-THSD7A antibodies. The extrarenal anti-THSD7A antibodies circulate into the glomerular capillaries and bind to THSD7A antigens located on podocytes. Besides, the tumor may alter the renal microenvironment that induces antigen conformational changes that allow binding of anti-THSD7A antibodies. (b) Pathogenicity of anti-THSD7A antibodies causing podocyte injury and proteinuria. The binding of anti-THSD7A antibodies to THSD7A on podocytes disrupts the slit diaphragm that allows albumin excretion into the urine, collapse of the actin cytoskeleton, and reduction of detachment from GBM. (c) The normal structure of podocyte foot processes. THSD7A that is present in foot processes closest to the slit diaphragm is involved in the stabilization of the slit diaphragm of mature podocytes, which forms the final barrier to albumin permeation. Abbreviations: APC: antigen-presenting cell; TCR: T cell receptor; MHC: major histocompatibility complex; GBM: glomerular basement membrane.

The possibility that antipodocyte antibodies alter podocyte function or induce podocyte injury in the absence of detectable complement activation is illustrated by THSD7A studies. In 2016, Tomas et al. [32] first isolated anti-THSD7A antibodies from patients and administered them to mice. They found that binding of autoantibodies to THSD7A on mouse podocytes resulted in the onset of proteinuria and a histopathological pattern that is typical of MN. Unlike previous findings that the activation of the complement system was responsible for podocyte damage, they detected that C3 and C5b-9 deposits were absent in these mice, whereas the histopathologic pattern of MN was initiated with induction of proteinuria at 3 days. In a subsequent study, they also could not detect membrane attack complex in mice with podocyte injury after the injection of rabbit anti-THSD7A antibodies [33]. Further *in vitro* experiments showed that the anti-THSD7A antibody directly induced cytoskeleton rearrangement in THSD7A-expressing glomerular epithelial cells and activated focal adhesion-mediated signaling. These findings strengthen the preceding conclusion that complement activation is not vital in the initiation of podocyte injury and that anti-THSD7A antibodies can directly affect podocyte integrity *in vitro*, causing cell damage and proteinuria. Remarkably, knockdown of thsd7aa—the THSD7A ortholog—in zebrafish larvae interfered with podocyte differentiation and impaired glomerular filtration barrier integrity, suggesting an important role of THSD7A in normal podocyte function [33]. In a recent study, Herwig et al. [34] investigated the temporal expression, spatial expression, and biological function of THSD7A in podocytes to provide insights into the effect of THSD7A antibody in MN. THSD7A was found to be present in foot processes (FPs) closest to the slit diaphragm that connects interdigitating podocytes and prevents most proteins from entering the urinary space; its expression begun with glomerular vascularization during the capillary loop stage [35] (Figure 2(c)). In addition, they found that phenotypically, THSD7A was expressed in distinct membrane domains, such as TAPs, resulting in augmented podocyte adhesion and stabilization of podocyte cell dynamics. Addition of anti-THSD7A antibodies in situ disrupted the flexible nonclogging barrier to proteins by mechanical alteration of the slit diaphragm, which is specific to the three-layered glomerular filter. Adhesion of the podocyte foot process to the glomerular filtration membrane was reduced by perturbation of the biological function of THSD7A. Both could destabilize the dynamics of FP and slit diaphragms, resulting in FP effacement and proteinuria, which are hallmarks of MN (Figure 2(b)).

## 4. Extrarenal Autoantigen Exposure

The location of autoantigens initially exposed to the immune system and how the immune response initiates the production of autoantibodies against podocytes and causes damage to podocytes remain controversial. Under normal circumstances, antigen-presenting cells or circulating T cells cannot directly contact autoantigens expressed in podocytes [36]. Notably, both PLA2R and THSD7A have a wide tissue distribution, including the kidneys. The extrarenal expression of antigens provides a basis for extrarenal autoantibody production. We hypothesize that primary exposure to autoantigens may occur in human organs other than the kidneys. PM2.5, gene expression, and tumors significantly facilitate the exposure of extrarenal antigens to the immune system and might account for the onset of MN.

### 4.1. Discharge of PLA2R: The Consequence of PM2.5 in iMN

**Figure 3:**
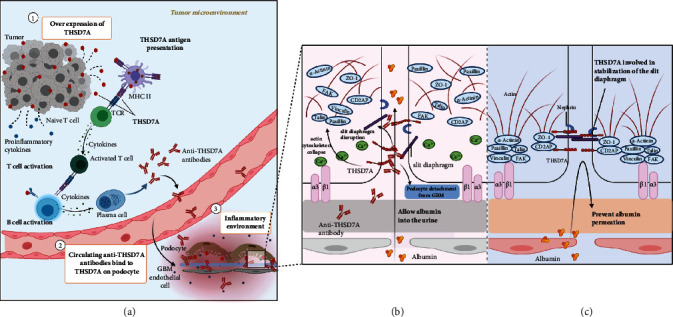
The hypothetical pathogenesis model of PM2.5-associated idiopathic membranous nephropathy (iMN). (a) PM2.5 induces extrarenal anti-PLA2R antibody production. Inhalation of PM2.5 results in the accumulation and activation of alveoli macrophages and neutrophils. PLA2R that is presented on these cells can be discharged into the inflammatory space, when neutrophil extracellular traps (NETs) and macrophage extracellular traps (METs) are released. Inflammation enhances the immunogenicity of the autoantigen and affects the antigen processing capacity of antigen-presenting cells (APCs), which contributes to the autoimmune response. We hypothesize that the PLA2R antigen may be captured by mature APCs, which become accessible producing anti-PLA2R antibodies. (b) The in situ immune complexes are initiated by binding of extrarenal anti-PLA2R antibodies to endogenous PLA2R in the glomeruli. PM2.5 can also cause renal injury and alter renal microenvironment, which may affect the molecular conformation of PLA2R antigen on the podocytes that is necessary for anti-PLA2R antibodies to bind. Abbreviations: GBM: glomerular basement membrane.

Given that people who are exposed to high levels of PM2.5 in the province in China for a long time have an increased risk of MN, the effect of PM2.5 on glomerulopathy should be considered [15]. Although PLA2R is highly expressed in the kidney, it is also present in neutrophils [37], alveolar macrophages [38], airway epithelial cells, and submucosal epithelial cells in humans [39]. The lung, which has a large surface area, is vulnerable to environmental factors. Inhalation of PM2.5 can cause lung inflammation that results in the accumulation of inflammatory corpuscles in the airway or alveoli. Activated neutrophils and macrophages release neutrophil extracellular traps and macrophage extracellular traps, and PLA2R can also be discharged into the systemic circulation, initiating an autoimmune response [40] (Figure 3).

In addition, we hypothesize that PM2.5 exposure contributes to aberrant immune processing by activating antigen presentation and augmenting autoimmune responses. Experimental studies have shown that air pollution exposure and oxidative stress induce antigen-presenting cell (APC) maturation [41–45] and then equip them with antigen peptide MHC molecular complex required for the activation of T cell receptors. Interestingly, most of the cells involved in inflammation can also present antigens [46]. Of special relevance here is the observation that air pollutants (especially fine particulates) stimulated the production of cytokines, immunoglobulins, and immune complexes and led to immune dysfunction, which is related to the pathogenesis of some glomerular diseases [47–49]. In addition, PM2.5 can be considered a foreign body that induces activation of cellular immunity in the lungs. Thus, it is plausible that PM2.5-induced exposure of PLA2R outside the kidneys could increase the incidence of MN.

### 4.2. Facilitating Antigen Presentation: Genetic Susceptibility in MN

Genetic analysis has opened new ways to investigate the link between PLA2R-associated MN and T cell epitopes [50]. It is necessary to reveal the initial step of MN by understanding the interaction between the HLA-D and PLA2R1 loci in PLA2R-related MN. In 1979, iMN was already reported to be strongly associated with HLA-DR3 [51] and this was confirmed in subsequent studies. Owing to the development of genome-wide association studies for the identification of PLA2R, considerable progress has been made in the understanding of the molecular genotypes, which refined the locus to two series of alleles, HLA-DQAI and PLA2R1 [52]. The most significant locus was on chromosome 6, centered on HLA-DQA1, and homozygosity for the lead risk alleles increased the odds ratio (OR) for MN by 20-fold. The other strong signal came from PLA2R1, in which homozygosity increased the OR for MN by 4-fold. Meanwhile, because of the interaction between the two loci, homozygosity in both risk alleles increased the OR by 80-fold. Although the risk allele in PLA2R1 is intronic, it is unlikely to change the autoreactivity of PLA2R. A follow-up study by sequencing the coding sequence and splice sites of PLA2R1 in 95patients with iMN identified no amino acid variations that were structurally specific to MN [53]. Because genetic variations in the amino acid sequence are common, it is hypothesized that the genetic susceptibility for iMN is conferred by single-nucleotide polymorphisms that might influence the expression levels or site of PLA2R in the presence of risk HLA alleles [54].

**Figure 4:**
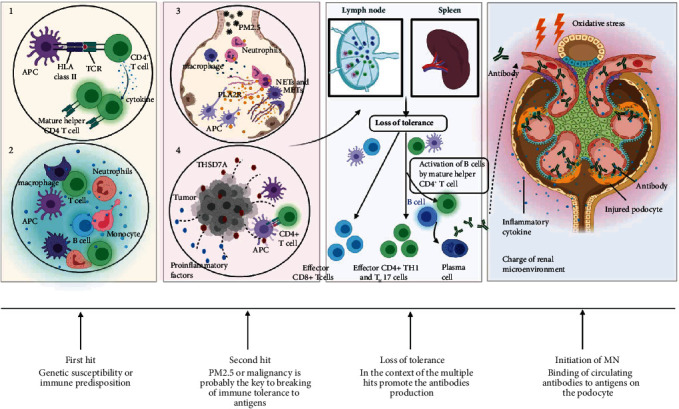
A multihit mechanism for the development of membranous nephropathy (MN). Genetic susceptibility or immune predisposition is thought to be involved in the ‘first hit' that probably drives the initial development of MN. Furthermore, PM2.5 or malignancy probably emerges as “second hit” that exerts an additive effect on the activation of the immune response. In the context of multihit that causes loss of immune tolerance, T helper 1 (T_H_1) and 17 (T_H_17) cells are essential for autoantibody production. Once such an autoimmune response is established, proinflammatory cytokines would act to exacerbate the ongoing response. Besides, inflammation or pathogenic factors alter renal microenvironment, injure podocytes, and enhance the immunogenicity of autoantigens, which contribute to the development of MN. During the development of MN, circulating antibodies binding to the podocyte may be a perfect storm, rather than a straight forward conformeropathy. ① Risk allele associated with MN. ② Immune predisposition. ③ PM2.5 induces extrarenal phospholipase A2receptor (PLA2R) exposure to immune cells. ④ Malignancy induces thrombospondin domain-containing 7A (THSD7A) overexpression. Abbreviations: APC: antigen-presenting cell; HLA: human leukocyte antigen; TCR: T cell receptor; NETs: neutrophil extracellular traps; METs: macrophage extracellular traps.

Based on these initial studies, several studies have confirmed the presence of risk alleles in or near HLA-DQA1 [55–58] and found an association between the specific HLA − DQA1^∗^0501 allele and the presence of circulating anti-PLA2R antibodies [56, 57, 59]. However, the situation is more complex; the degree of linkage disequilibrium of the HLA locus is high and several studies found that a risk signal could lie in the HLA-DRB locus. Cui et al. [60] identified independent risk alleles in DRB1∗1501 and DRB1∗0301, suggesting certain amino acid positions in the major histocompatibility complex (MHC) DR*β*1 chain facilitate interactions with T cell epitopes of PLA2R. HLA class II genetic restriction controls specific peptides of PLA2R presenting to T cells to drive active B cells for high-affinity autoantibody production (Figure 4, ①). Another study by Le et al. [61] not only identified the most significant risk allele in DRB1∗1501 but also revealed another independent significant risk allele in DRB3∗0202. Of note, one of these two HLA haplotypes was also found in 44% of healthy controls, demonstrating that the presence of high-risk alleles may be necessary but not sufficient for the development of PLA2R-related MN. In addition to MN, HLA-DQA1 risk alleles are significantly associated with lupus nephritis, type 1 diabetes, and focal segmental glomerulosclerosis in a German population [55].

In summary, the particular podocyte antigens targeted by autoantibodies may be involved in the genetic make-up. Genetic variants may change the molecular conformation of the antigens to expose new conformational epitopes or cryptic sites to enable processing to linear T cell peptides that facilitate the recognition of antigens by the immune system. However, variants in the coding regions and HLA class II genes are common and gene expression alone is not enough to explain why some individuals develop MN, whereas others do not or some develop late onset disease [62]. Alternatively, we propose that the genetic susceptibility to iMN may not depend on the concurrence of PLA2R1 risk alleles but on the cooccurrence of HLA-D and other external triggers. Therefore, further studies are needed to identify other potential mechanisms affecting antigen conformation, and aberrant immune processing could lead to the production of anti-PLA2R antibodies in genetically predisposed patients with HLA and/or PLA2R1 risk variants.

### 4.3. Overexpression of THSD7A: The Role of Tumor in iMN

The association between MN and cancer has been controversial, unlike PLA2R, which has been suggested to be a tumor suppressor [63], and patients with THSD7A-associated MN have a significantly increased risk of malignancy. Hoxha et al. [64] and Hanset et al. [65] found that 20% and 50% of patients with THSD7A-associated MN were diagnosed with malignancy on further work-up, respectively. In addition, two typical cases, one with metastases of endometrial carcinoma and the other with gallbladder tumor, further explain the potential molecular mechanism of disease induction in this setting [64, 66]. In both cases, THSD7A was found to be expressed in follicular dendritic cells of lymph nodes with metastatic infiltration. Assessment of the cancerous tissues showed increased THSD7A mRNA levels and elevated THSD7A protein expression, suggesting that THSD7A was actively synthesized by cancer cells. In addition, several studies found that anti-THSD7A antibodies were correlated with malignancy, benign tumors, and neurological disease [66–68], which increases the possibility of extrarenal anti-THSD7A immunization. It is worth noting that upon tumor remission, even without the use of immunosuppressive agents, kidney disease can also be alleviated. Stahl et al. [69] reported that THSD7A, as a new tumor antigen, plays a potential role in human cancer. The finding that the expression of THSD7A differed according to the clinical stage and differentiation degree of various cancers suggests that THSD7A is involved in vascular invasion, cancer progression, metastasis, and angiogenesis mechanisms that support tumor growth.

Tumor growth requires an increased intratumoral blood supply that promotes angiogenesis, in which THSD7A, a tumor-associated antigen, is overexpressed, allowing regional exposure of the autoantigen to the immune system (Figure 2(a)). If this hypothesis is confirmed, it is necessary to reconsider the concept of primary versus secondary MN.

## 5. Antigen Conformational or Expression Changes

In the inflammatory state, the production and accumulation of reactive oxygen species (ROS) exceeds the ability of cells to clear oxides, and the imbalance between the oxidation and antioxidant systems leads to the development of oxidative stress. Under conditions of intense oxidative stress, disulfide bonds are formed; however, most proteins do not form disulfide bonds in a reducing environment [70]. It is worth noting that epitopes in both PLA2R and THSD7A require intact disulfide bonds to maintain their spatial structure and bioactivity [3, 4]. Thus, the antigenicity of PLA2R and THSD7A requires additional conditions. If oxidative stress is able to influence the development of disulfide bonds, a synergistic effect of exposure to these antigens or overexpression under pathological conditions can be expected.

### 5.1. Change in Conformation of Intrarenal Antigens

Because the lungs are directly exposed to the external world, pulmonary inflammation and airway injury caused by PM2.5 are highly prevalent [71]. PM2.5 is the carrier of toxic substances, and polycyclic aromatic hydrocarbons and transition metals adsorbed in it can also directly produce ROS, exacerbating inflammation [72]. Moreover, it has been reported that levels of superoxide radicals, H_2_O_2_, and malondialdehyde increased and superoxide dismutase decreased in tissues and cells, suggesting that PM2.5 upregulated oxidative stress [73]. Under this strongly oxidizing environment, disulfide bonds can be formed in cytoplasmic proteins [74]. Therefore, when the lungs are exposed to PM2.5, the stability of PLA2R epitopes may facilitate selection of B cells for generation of captured antigens on MHC molecules to present to T cells, which is an important step in the initiation of the autoimmune mechanism in MN. Then, activated B cells differentiate into plasma cells with the assistance of Th cells and produce antibodies against the different epitopes of the PLA2R antigen [21]. In addition, PLA2R, following a pH-dependent configuration change, may adopt an extended configuration in the extracellular domain when the microenvironment is altered. Thus, the three main epitopes located in the CysR, CTLD1, and CTLD7 domains may be more accessible in the extended conformation of PLA2R that would otherwise not be recognized by the immune system, triggering epitope spreading.

Inflammation is one of the pathways activated because of environmental stimuli as well as cancer, which is linked to the pathogenesis of MN. Recent studies have demonstrated that cancer initiation and progression are linked to inflammation and oxidative stress [75]. Inflammation is considered a hallmark of cancer in the tumor microenvironment. Indeed, cancer is viewed as a wound that does not heal [76], inducing persistent activation of inflammatory signals and releasing proinflammatory cytokines. Thus, cancer recruits inflammatory cells and stimulates them to generate ROS, reactive nitrogen intermediates, and cytokines [77, 78], that is, like a bridge, ROS plays a critical role in the close association between inflammation and cancer [79]. Therefore, during tumor development, upregulation of THSD7A and persistent expression of pathogenic epitopes triggers an immune response. In the first step, regionally expressed THSD7A, due to oncogenesis, is released and taken up by dendritic cells (DCs), which coordinate specific immune signals to lose peripheral tolerance toTHSD7A. Such immunogenic signals might include proinflammatory cytokines and factors, such as TNF-*α*, IL-1, IFN-*α*, and CD40L/CD40, to promote immunity. DCs present a THSD7A antigen peptide to T cells in the form of antigen peptide MHC molecular complex, resulting in the activation of cytotoxic T lymphocytes and helper T cells (Th) against the THSD7A antigen. Developed mature Th cells and numerous cytokines drive the differentiation of activated B cells into plasma cells, which produce high-affinity antibodies [80].

### 5.2. Change in Expression of Podocyte Target Antigens and Other Potential Antigens

Circulating antibodies produced by extrarenal antigen exposure can specifically bind to podocyte target antigens through endothelial cells and GBM. In addition, the anti-PLA2R1 and anti-THSD7A antibodies initially produced against nonpodocyte antigens exclusively bind with podocyte antigen epitopes determined by the presence of disulfide bonds in a nonreducing state [4]. The role of antigen conformational charges in extrarenal tissue has been suggested in anti-GBM disease [81]. Exactly as that in Goodpasture's disease, MN may also be considered an autoimmune “conformeropathy” involving pathogenic conformational changes. Interestingly, air pollution and malignancy both cause oxidative stress and inflammation, which trigger the release of secondary mediators. The resulting cytokines and oxygen free radicals may exert distant effects in other organs, such as the kidneys. A study in China confirmed renal injury after consecutive exposure to PM2.5 and revealed that it occurred via inflammatory cytokine and chemokine expression and reduced antioxidant activity [73]. Analysis of renal biopsies in THSD7A-associated MN with malignancy revealed that more inflammatory cells infiltrated the glomeruli in patients with no malignancy [64, 82]. Therefore, both PM2.5 and malignancy may contribute to the nonreducing state in the renal microenvironment, enabling circulating antibodies to bind to cryptic epitopes that may be hidden in healthy individuals.

Antibodies against cytoplasmic podocyte proteins, including *α*-enolase, aldose reductase, and manganese superoxide dismutase 2, have been detected in the sera of patients with primary and secondary MN [83, 84]. However, these cytoplasmic antigens are not accessible to circulating antibodies under normal conditions. Oxidative stress caused by PM2.5 and tumors may be a primary insult to podocytes, which is responsible for the novel membrane expression of cytoplasmic antigens, which triggers the formation of new antibodies [85, 86]. Although intriguing, the role of oxidative stress in the initiation and/or maintenance of the disease has not been firmly established.

## 6. Disparate Paths to the Same Disease

The initiation of MN is the result of a multihit mechanism. Based on the current understanding, we recognize the possibility that associated factors, including malignancy, chronic infection, or environmental factors, may represent a disease-precipitating “second hit” in a patient with genetic and immune predisposition to develop MN [87]. Through a large number of high-quality research [15, 64, 66, 69, 88], we discuss the similarities between PLA2R and THSD7A, focus on the effect of PM2.5 and tumor in MN, and allow to better identify the potential pathogenic factors in MN **(**Figure 4**)**.

IgG4 is the most prevalent subclass in most cases of PLA2R- and THSD7A-related MN. IgM > IgG3 > IgG1 > IgG2 > IgG4 is considered the temporal model sequence of Ig class switching in the germinal center reaction [89]. To note, in the progress of MN, Ig subclasses switch from IgG1 at the early stage to IgG4 at all later stages [90]. Induced by environmental stimuli or other causes, the PLA2R1 antigen and/or THSD7A antigen constant is exposed to extrarenal tissues. During prolonged disease activity, high-affinity IgG4 often formed by following repeated or long-term exposure to antigen [91–93]. Based on this understanding, we have further speculated that the occurrence of MN is due to prolonged exposure of some factors. PLA2R and THSD7A expressed in extrarenal tissues and presenting pathogenic epitopes are necessary to elicit autoimmune responses. It should be noted that both PM2.5 and malignancy may induce extrarenal antigen exposure to immune cells through conformational changes, molecular mimicry, or upregulated expression and directly damage the kidneys. Inflammation enhances the immunogenicity of autoantigens and affects the antigen processing capacity of APCs, which contributes to the autoimmune response. Undoubtedly, genetic susceptibility influences the development of MN, and other pathogenic factors exert an additive effect in determining whether the genetic potential is manifested [94]. Immunization can be augmented by the unmasking of hidden cryptic epitopes undergoing conformational changes upon exposure to environmental stimuli [95]. The MN may appear to be primary but in fact represent occult secondary disease. The discovery of other factors that promote antigen recognized by the immune system is worthy of further exploration and confirmation. In addition to PM2.5 and tumor being clearly related to the occurrence of MN, dysbiosis of the gut microbiota could be another factor. Indeed, the existing studies indicate that patients with MN exhibited gut microbial signatures distinct from healthy controls, which suggests the potential of gut microbiota as a contributor in the pathogenesis of MN [96, 97]. However, the direct evidence for the relationship between dysbiosis of the gastrointestinal flora and the development of MN is lacking and needs to be further enriched.

In addition, PLA2R and THSD7A expressed in podocytes play an indispensable role in maintaining the integrity of podocyte function, which may determine why the disease mainly affects podocytes and the pathological features are limited to the kidney. The location of autoantigenic proteins is restricted to the space between podocytes and the GBM. The clearance of immune complexes under the glomerular epithelium may be blocked; however, the bronchial epithelium is in direct contact with the environment and the cell turnover rate is high, which may promote the clearance of immune deposits through the sputum. Moreover, the biology of autoantigenic proteins in podocytes is another key precondition. Fresquet et al. [98] offered insights into the role of PLA2R in podocytes through PLA2R and the A2t complex. They found that PLA2R may be at the heart of actin cytoskeleton reorganization and tight junction assembly. *In vitro*, anti-PLA2R antibodies interfere with the adhesion of podocytes to collagen type IV in MN [99]. As mentioned above, THSD7A plays an important role in the integrity of the glomerular filtration barrier. Autoantibodies to THSD7A might alter the structural and functional permeability of the slit diaphragm to proteins.

In conclusion, the discovery of PLA2R and THSD7A as two autoantigens in MN has been a “game changer” that has fundamentally changed our approach toward diseases. However, many questions remain unsolved. In this review, we analyzed the similarities in the pathological mechanisms triggered by disparate antigens and their associated diseases resulting in the same renal phenotype to explore the triggers of MN. Further research is required to reveal the molecular pathogenesis of MN in the lungs and tumors and investigate innovative therapeutic strategies targeting MN-specific pathological mechanisms.
